# Evaluating the Therapeutic Impact of Immune Checkpoint Inhibitors in the Management of Brain Metastases from Non-Small Cell Lung Cancer

**DOI:** 10.3390/biomedicines14030566

**Published:** 2026-03-02

**Authors:** Keren Rouvinov, Rashad Naamneh, Alexander Yakobson, Arina Soklakova, Wenad Najjar, Mahmoud Abu Amna, Mohnnad Asla, Ashraf Abu Jama, Nashat Abu Yasin, Mhammad Abu Juda, Sofyan Abu Freih, Yotam Malek, Walid Shalata

**Affiliations:** 1 The Legacy Heritage Cancer Center, Dr. Larry Norton Institute, Soroka Medical Center, Beer-Sheva 84105, Israel; 2 Goldman Medical School, Faculty of Health Sciences, Ben-Gurion University of the Negev, Beer-Sheva 84105, Israel; 3 Neurosurgery Department, Soroka Medical Center, Beer-Sheva 84105, Israel; 4 Neurosurgery Department, Hadassah Hebrew University Medical Center, Jerusalem 91200, Israel; 5 The Ruth and Bruce Rappaport Faculty of Medicine, Technion, Haifa 31096, Israel

**Keywords:** non-small cell lung cancer, brain metastases, immune checkpoint inhibitors, intracranial objective response rate, intracranial disease control rate, overall survival

## Abstract

Background: Non-small cell lung cancer (NSCLC) patients often develop brain metastases (BMs), with an incidence rate of 25–40%. These metastases are associated with a poor prognosis. Although immune checkpoint inhibitors (ICIs) have revolutionized NSCLC treatment, their effectiveness against BMs remains not well-established. This is primarily because patients with active or symptomatic BMs have frequently been excluded from clinical trials. Methods: To address the lack of data, in this narrative review, we searched the available clinical guidelines and relevant studies. Databases, including PubMed, MEDLINE, Embase, Cochrane Central Register of Controlled Trials (CENTRAL), and Web of Science, were searched for clinical studies published up to June 2025. The article included studies of NSCLC patients with BMs who were treated with ICIs and had reported central nervous system (CNS)-specific outcomes. Results: This article found that ICIs show significant intracranial activity. This activity is particularly notable in select NSCLC patients with asymptomatic BMs and high Programmed Death-Ligand 1 (PD-L1) expression. Conclusions: More research is needed to fully understand the efficacy of ICIs in treating BMs. Future prospective studies must include patients with active and symptomatic BMs to validate these findings and provide a clearer picture of ICI efficacy in this patient population.

## 1. Introduction

BMs are a prevalent and devastating complication in patients diagnosed with NSCLC, affecting approximately 20–40% of individuals. Autopsy studies have reported an even higher incidence, reaching up to 52%. These intracranial lesions are a major determinant of patient morbidity and mortality, profoundly diminishing quality of life and leading to a dismal prognosis [1]. The median survival following a BM diagnosis typically ranges from 3 to 11 months, with variability influenced by factors such as the number of intracranial lesions, response to treatment, and the primary tumor’s origin [1,2,3].

Conventional systemic therapeutic approaches have demonstrated limited efficacy. This is primarily due to the inherent challenges posed by the blood–brain barrier (BBB), which restricts the penetration of many systemic agents, and the distinct biological characteristics that differentiate BMs from their primary lung counterparts. The unique tumor microenvironment (TME) within the brain also contributes to the complexity of treatment, often exhibiting an immunosuppressive nature that further impedes therapeutic success [4,5,6].

The landscape of advanced NSCLC treatment has been fundamentally transformed by the introduction of ICIs. These agents, which target crucial immunological pathways such as programmed death protein-1 (PD-1), its ligand (PD-L1), and cytotoxic T-lymphocyte-associated protein 4 (CTLA-4), function by disinhibiting the host immune response against cancer cells. This mechanism has led to remarkable improvements in OS and response rates for patients with advanced NSCLC [6,7,8,9].

Despite these systemic successes, the application and efficacy of ICIs specifically in NSCLC patients with BMs have historically been subjects of considerable uncertainty and limited investigation [8,9,10]. The prevailing assumption of the brain as an “immune-privileged” site, coupled with concerns regarding drug penetration across the BBB and potential neurological toxicities, led to the widespread exclusion of these patients from early ICI clinical trials. In addition, the frequent need for corticosteroids in patients with symptomatic brain metastases may both attenuate the efficacy of immune checkpoint inhibitors and contribute to their exclusion from clinical trials.

Given the severe prognosis associated with NSCLC BMs and the inherent limitations of conventional treatments, there is an urgent and critical need to rigorously evaluate and optimize innovative systemic therapies like immunotherapy for this vulnerable patient population. Emerging scientific understanding has challenged the long-held notion of the brain as an immune-isolated compartment [1,2,3,4]. Recent evidence increasingly indicates that the brain is, in fact, an immunologically active organ capable of initiating and regulating immune responses, thereby supporting the biological rationale for ICI activity within the CNS [1,2,3,4,8,9,10,11,12]. This evolving understanding underscores the imperative to thoroughly investigate and integrate immunotherapy into the multidisciplinary management of NSCLC BMs, aiming to improve both survival and neurological outcomes.

## 2. Searching Strategy

This narrative review synthesizes the existing literature on ICIs in NSCLC with BMs. A structured but non-systematic search of PubMed, MEDLINE, Embase, the Cochrane Central Register of Controlled Trials (CENTRAL), and Web of Science was performed using combinations of the following keywords: “NSCLC,” “brain metastases,” “immunotherapy,” “checkpoint inhibitors,” “immune checkpoint inhibitors,” “PD-1,” and “PD-L1.” Studies published through June 2025 were considered. Relevant articles were selected based on clinical relevance and focus on patients with histologically confirmed NSCLC and documented brain metastases treated with immune checkpoint inhibitors (including nivolumab, ipilimumab, pembrolizumab, and atezolizumab), reporting outcomes such as safety, intracranial objective response rate (iORR), intracranial disease control rate (iDCR), or survival. Case reports and very small case series (fewer than five patients), as well as review articles, were excluded to maintain clinical applicability. Study selection was guided by relevance to the scope of this review.

## 3. Current Evidence from the Literature

The landscape of immunotherapy for NSCLC BMs is increasingly supported by a growing body of high-level evidence, including several reviews and meta-analyses. These comprehensive syntheses represent a crucial advancement, moving beyond individual trial data to provide a more robust understanding of ICI efficacy and safety in this challenging patient population. Evidence suggests a positive effect of immunotherapy on NSCLC patients with BMs [12,13,14,15].

One notable systematic review and NMA identified 11 studies, encompassing 1437 participants, that investigated the efficacy and safety of various immunotherapy types for NSCLC BMs [12,13,14,15]. Another systematic review and meta-analysis, known as the META-L-BRAIN Study, specifically examined ICIs as a single treatment modality for untreated BMs, incorporating data from 12 studies and 566 individuals. Further contributing to this evidence base, a comprehensive systematic review of 14 randomized controlled trials (RCTs), involving 9089 patients, specifically addressed whether the presence of asymptomatic BMs influenced the overall efficacy of immunotherapy in lung cancer [12,13,14,15].

### 3.1. Key Findings from Meta-Analyses

The aggregated data from these analyses provide critical insights into the comparative efficacy and safety of immunotherapy in NSCLC brain metastases, primarily in comparison with chemotherapy-based regimens.

#### 3.1.1. Comparative Efficacy

Pembrolizumab has emerged as a particularly promising immunotherapy for NSCLC patients with BMs. It demonstrated a significant improvement in OS and a better effect in improving the ORR [9,10,12,13,14].

The combination of nivolumab and ipilimumab also showed a notable improvement in OS, suggesting it as a viable alternative for enhancing survival outcomes [9,10,12,13,14].

#### 3.1.2. Intracranial Activity

Anti-programmed cell death protein-1 (anti-PD-1) therapy has demonstrated discernible activity within the CNS. A meta-analysis reported an intracranial objective response rate of 16.4% (95% CI: 9.8–24%) and an iDCR of 45% (95% CI: 33.4–56.9%) for untreated BMs. Importantly, this analysis found no significant difference in iORR or iDCR between patients whose BMs were treated with radiotherapy prior to ICI initiation and those who received ICI as a monotherapy [9,10,12,13,14].

#### 3.1.3. Influence of BM Status

Immunotherapy generally conferred a significant survival advantage to patients with BMs, evidenced by an OS hazard ratio (HR) of 0.72 (95% CI, 0.58–0.90; *p* = 0.004) and a progression-free survival (PFS) HR of 0.68 (95% CI, 0.52–0.87, *p* = 0.003). A critical finding from this review was that the presence of BMs did not significantly influence the overall efficacy of immunotherapy. The pooled ratios of OS-HRs and PFS-HRs reported in BM patients versus non-BM patients were 0.96 and 0.97, respectively (*p* > 0.7), indicating comparable benefits across both groups [9,10,12,13,14,15,16,17].

#### 3.1.4. Safety Profile

The NMA observed no significant difference in the incidence of AEs when comparing immunotherapy regimens with chemotherapy, suggesting a generally manageable safety profile for ICIs in this population [14,15].

The accumulation of multiple dedicated meta-analyses and systematic reviews specifically addressing immunotherapy in NSCLC BMs marks a pivotal shift in the field (Table 1). Historically, patients with BMs were often excluded or significantly underrepresented in pivotal clinical trials due to concerns about drug penetration across the BBB, potential for diminished efficacy with concurrent steroid use, and the risk of hyperprogression. The emergence of sufficient primary data to enable such high-level evidence synthesis directly challenges these historical limitations. This progression from an era of data scarcity to one of growing confidence in ICI activity within the CNS signifies a profound re-evaluation of treatment guidelines and trial designs. The field is now moving towards a more inclusive approach, transforming what was once a largely unaddressed patient population into a central focus of active investigation and therapeutic advancement, ultimately aiming to improve clinical outcomes for these individuals.

## 4. Efficacy and Safety of Immunotherapy Agents in NSCLC BMs by Types of Immunotherapy Agents and Their Efficacy

### 4.1. Pembrolizumab

Pembrolizumab has emerged as a leading agent, identified as the most promising immunotherapy for NSCLC patients with BMs. An NMA demonstrated its significant positive effect on (OS and overall response rate ORR [10]. Furthermore, a phase 2 trial specifically investigating pembrolizumab in NSCLC patients with untreated, asymptomatic BMs reported an iORR of 29.7% (95% CI, 15.9–47.0) [19].

### 4.2. Nivolumab + Ipilimumab

This combination exhibited an improvement in OS, positioning it as a valuable alternative choice for improving survival outcomes in NSCLC BMs. A post hoc analysis of the Checkmate 227 trial suggested comparable efficacy of this dual-ICI therapy irrespective of the presence of baseline BMs [11,20].

### 4.3. ICI + Chemotherapy

Pooled analyses of trials combining pembrolizumab with chemotherapy (e.g., KEYNOTE-021, KEYNOTE-189, and KEYNOTE-407) indicated improved survival outcomes regardless of whether BMs were present at baseline [21,22,23,24].

### 4.4. Treatment Outcomes

The therapeutic impact of immunotherapy in NSCLC BMs is reflected across several key clinical endpoints:

#### 4.4.1. OS

Significant improvements in OS have been observed with both pembrolizumab monotherapy and the nivolumab + ipilimumab combination. Overall, immunotherapy has consistently conferred a survival advantage to patients with BMs, with a reported HR for OS of 0.72 [13].

#### 4.4.2. PFS

Patients with BMs also experienced a PFS benefit with immunotherapy; in the same meta-analysis, the pooled PFS HR was 0.68 [13,25,26].

#### 4.4.3. Intracranial Response Rates (iORR, iDCR)

Anti-PD-1 therapy has demonstrated notable intracranial activity. One meta-analysis reported an iORR of 16.4% and an iDCR of 45% for untreated BMs. A phase 2 trial of pembrolizumab further substantiated this, showing an intracranial ORR of 29.7% in patients with untreated, asymptomatic BMs [12,19].

### 4.5. Distinguishing Efficacy in Treated vs. Untreated Brain Lesions

The efficacy of immunotherapy in NSCLC BMs can vary depending on whether the lesions have been previously treated.

#### 4.5.1. Untreated BMs (ICI Monotherapy)

Emerging data suggest promising activity for ICIs as monotherapy in select patients with untreated BMs. A phase 2 trial of pembrolizumab reported an intracranial response in 29.7% of patients with untreated, asymptomatic BMs. Similarly, Arm M of the Checkmate 012 trial observed a 16.7% intracranial response rate in NSCLC patients with asymptomatic, untreated BMs. These findings indicate that ICIs can be effective as a single treatment for active BMs in carefully selected patients [14,27].

#### 4.5.2. Pretreated BMs (ICI Monotherapy or Combinations)

Pooled analyses of various trials, including those for pembrolizumab (KEYNOTE-001, -010, -024, -042) [16,28,29,30], nivolumab (Checkmate 063, 017, 057) [31,32,33], and atezolizumab (OAK) [17], consistently demonstrated improved survival with ICIs regardless of baseline BMs presence, specifically in patients whose brain lesions were previously treated and stable. Similar efficacy was also observed for dual-ICI therapy (ipilimumab plus nivolumab) in patients with treated and stable BMs.

While ICIs demonstrate promising activity in both previously treated and untreated BMs from NSCLC, a critical nuance in the observed efficacy pertains to the patient selection criteria for trials involving untreated lesions (Table 2).

The reported intracranial responses in this subgroup are predominantly observed in asymptomatic patients who do not require concurrent steroid therapy. This observation is significant because it suggests that the reported benefits may not be broadly generalizable to all patients with untreated BMs, particularly those with symptomatic or larger lesions that necessitate corticosteroid administration [33,34,35,36,37,38,39,40,41]. The underlying clinical challenge arises from the immunosuppressive effects of corticosteroids, which are often indispensable for managing cerebral edema and neurological symptoms associated with BMs but can potentially diminish the anti-tumor efficacy of ICIs [33,34,35,36,37,38,39,40,41]. This creates a therapeutic dilemma: balancing the immediate need for symptomatic control with the desire to optimize immunotherapy response. Therefore, developing strategies to mitigate steroid dependency or to enhance ICI efficacy even in the presence of corticosteroids represents a crucial unmet need in the management of NSCLC BMs.

## 5. The Role of PD-L1 Expression in Predicting Treatment Outcomes

### 5.1. Correlation with ICI Therapy Efficacy

PD-L1 expression is widely recognized as a significant predictor of the success of ICIs in various cancers, including NSCLC with brain dissemination. Higher levels of PD-L1 expression are generally associated with improved OS, a correlation that holds true irrespective of the presence of BMs [40,42,43,44].

Specific clinical observations underscore this relationship. Preliminary results indicated that NSCLC patients with BMs who expressed more than 1% of the PD-L1 marker demonstrated a potential benefit from pembrolizumab treatment, with 3 out of 7 (43%) evaluable patients showing an intracranial response. Furthermore, treatment with first-line ICI was observed to be more beneficial when tumor PD-L1 levels exceeded 50%, with no reported deaths directly attributable to CNS disease in this subgroup. In patients with BMs who received a combination of radiation therapy and ICIs, increasing levels of PD-L1 expression were directly associated with improved survival outcomes [40,43,44,45,46,47]. For instance, OS rates were notably higher for patients with PD-L1 expression of 50–89% (29.5 months) and ≥90% (33.1 months), compared to those with PD-L1 < 1% (11.8 months) [43,44,45,46].

### 5.2. Methods of PD-L1 Assessment

The assessment of PD-L1 expression is a crucial component in guiding ICI therapy, and it typically involves specific scoring methodologies:

#### 5.2.1. Tumor Proportion Score (TPS)

This method is commonly employed for evaluating PD-L1 expression in advanced NSCLC. TPS quantifies the percentage of viable tumor cells that exhibit partial or complete membrane staining for PD-L1 at any intensity. The result is reported as a percentage ranging from 0% to 100%. For a specimen to be considered adequate for PD-L1 evaluation via TPS, a minimum of 100 viable tumor cells on the stained slide is required [45,46,47,48,49,50,51].

#### 5.2.2. Combined Positive Score (CPS)

CPS is utilized for assessing PD-L1 expression in various other cancer types, including metastatic or unresectable, recurrent head and neck squamous cell carcinoma, advanced HER2-negative gastric or gastroesophageal junction adenocarcinoma, and advanced triple-negative breast cancer. This scoring method evaluates the number of PD-L1-staining cells (which includes tumor cells, lymphocytes, and macrophages) relative to the total number of viable tumor cells. Although the calculated CPS can theoretically exceed 100, the maximum reported score is defined as CPS 100. Similar to TPS, a minimum of 100 viable tumor cells is necessary for adequate evaluation. In NSCLC, PD-L1 expression is typically assessed using Tumor Proportion Score (TPS), whereas Combined Positive Score (CPS) is primarily used in other tumor types and is included here only for methodological context [45,46,47,48,49,50,51].

#### 5.2.3. Cut-Off Values

Across studies, specific cut-off values for PD-L1 staining of tumor cells are frequently used to categorize expression levels: negative (<1%), weakly positive (1–49%), and strongly positive (≥50%). It is important to note that the determination of PD-L1 positivity is influenced by the specific assay utilized, the algorithm applied, the defined cut-off point, and the metastatic site where the PD-L1 status is evaluated [45,46,47,48,49,50,51].

### 5.3. Concordance and Challenges in PD-L1 Testing

While PD-L1 expression serves as a key biomarker, its application in the context of BMs is complicated by several factors:

#### 5.3.1. Discordance

A meta-analysis revealed a 19% disagreement in PD-L1 expression between primary lung tumors and their BMs, with BMs generally exhibiting lower levels of expression. Concordance rates reported across different studies vary significantly, ranging from 62.5% to 81%. Some investigations further suggest that brain metastatic tumors tend to have lower PD-L1 expression and fewer tumor-infiltrating lymphocytes (TILs) compared to primary lung tumors [48].

#### 5.3.2. Contradictory Findings

The prognostic value of PD-L1 for BM progression is not uniformly consistent across all studies. PD-L1 expression is widely used in clinical practice to guide treatment selection in advanced NSCLC (including decisions between single-agent immunotherapy and chemo-immunotherapy), but its predictive value is imperfect, particularly in patients with brain metastases. One retrospective study found no statistically significant association between high PD-L1 expression (≥50%) and a lower progression or development of BMs when compared to low expression (≤1%) [51]. This finding appears to contradict a 2018 study by Takamori et al. that suggested patients with BMs expressing PD-L1 (≥5%) had lower OS. Furthermore, another study observed that patients with low PD-L1 expression had better PFS and OS, which runs counter to the general trend of higher expression correlating with better outcomes [42,43,44,45,46,47,48,49,50,51,52,53,54,55,56].

While PD-L1 expression is a well-established and generally effective biomarker for predicting response to ICIs therapy in NSCLC, its utility in the specific context of BMs is complicated by significant biological and practical challenges (Table 3). The reported discordance in PD-L1 expression between primary lung tumors and their BMs, as well as the variability in concordance rates across studies, suggests that the brain’s unique immune microenvironment may lead to different biomarker profiles compared to extracranial sites. This inherent heterogeneity, combined with the formidable practical difficulties in obtaining BM tissue for direct PD-L1 assessment, implies that relying solely on primary tumor PD-L1 status may be insufficient or even misleading for guiding intracranial treatment decisions. This situation necessitates a critical re-evaluation of current biomarker strategies and drives the urgent need for more accurate, accessible, and CNS -specific predictive tools that can more reliably inform therapeutic choices for patients with NSCLC BMs.

## 6. Impact of Steroid Use on Immunotherapy Efficacy

The concurrent use of systemic corticosteroids presents a significant concern in the context of ICI therapy for BMs. Corticosteroids can diminish the efficacy of ICIs due to their broad immunosuppressive effects [55,56,57,58,59]. For patients presenting with large or symptomatic BMs, rapid local control is often paramount, necessitating the immediate use of steroids and upfront local CNS therapy (such as surgery and/or radiation therapy) in a multidisciplinary setting. In such cases, ICI treatment should ideally be paused until steroid doses can be tapered to mitigate the potential for reduced immunotherapy effectiveness [56,57,58,59].

Despite growing evidence of immunotherapy’s potential in NSCLC BMs, a substantial number of ongoing and pivotal trials continue to maintain stringent exclusion criteria for patients with active, untreated, or symptomatic BMs (Table 4). This includes trials like KEYNOTE-189 [59], CheckMate 227 [20], IMpower150 [60] CodeBreaK 202 [61] and SUNRAY-01 [62]. While some newer studies, such as PERLA [63] and NCT02886585 [64], demonstrate a more inclusive approach by allowing asymptomatic or stable treated BMs, the persistent exclusion of the most challenging patient subgroups—those with extensive, symptomatic, or rapidly progressing untreated lesions—means that robust, prospective evidence for these high-need populations remains critically scarce. In the key trials summarized in Table 4 (*n* = 9), steroid exclusion/restriction was explicitly reported in 6 studies (including 1 study requiring patients to be steroid-free and 5 using steroid-related restrictions), with 4 studies specifying thresholds of >10 mg/day prednisone-equivalent or >2 mg/day dexamethasone-equivalent. Asymptomatic brain metastases were included in 4 studies, whereas symptomatic brain metastases were not clearly included in any study. This enduring gap in clinical trial design compels clinicians to often rely on extrapolated data or retrospective analyses when making treatment decisions for these complex patients. This situation underscores the urgent and ongoing need for dedicated trials specifically designed to address the unique challenges and therapeutic requirements of these specific, complex scenarios, ensuring that evidence-based guidelines can be developed for all patients [20,59,60,61,62,63,64].

## 7. Recent Advancements and Emerging Biomarker-Driven Approaches

The field of immunotherapy for NSCLC BMs is undergoing rapid evolution, marked by the development of novel agents and sophisticated biomarker strategies aimed at overcoming existing challenges and enhancing therapeutic precision. Beyond conventional PD-1/PD-L1 and CTLA-4 inhibitors, several new classes of agents are showing promise:

### 7.1. Novel Immunotherapy Agents

#### 7.1.1. LAG-3 Inhibitors

Lymphocyte-activation gene 3 (LAG-3) is another immune checkpoint that, when inhibited, can enhance anti-tumor immunity. Preliminary results from the RELATIVITY-104 trial indicated a benefit from combining nivolumab with relatlimab (an anti-LAG3 antibody) and chemotherapy [65].

#### 7.1.2. B7-H3 Targeting

B7-H3, a member of the B7 family of immune checkpoint ligands, is often upregulated in BMs, where it contributes to an immunosuppressive microenvironment and can remodel tumor vasculature. Preclinical studies have shown that B7-H3 antibody-mediated targeting can reverse vascular leakiness and enhance CD8+ cytotoxic T-cell infiltration in mouse models, suggesting a promising therapeutic avenue [66].

### 7.2. Bispecific Antibodies

These innovative molecules are designed to simultaneously target two distinct antigens, offering the potential for enhanced efficacy and broader anti-tumor activity [67].

#### 7.2.1. Cadonilimab (AK104)

This novel bispecific antibody is currently under investigation in the COMPASSION-01 trial (NCT03261011) for its activity across diverse solid tumors, including metastatic NSCLC [68].

#### 7.2.2. Ivonescimab

This bispecific antibody uniquely targets both PD-1 and VEGF (vascular endothelial growth factor). The HARMONi-2 trial (NCT05499390) has suggested a PFS benefit in NSCLC patients with BMs treated with ivonescimab compared to pembrolizumab, highlighting the potential of dual targeting in the CNS microenvironment [69].

### 7.3. Antibody-Drug Conjugates

ADCs represent a targeted chemotherapy approach, combining the specificity of a monoclonal antibody with a potent cytotoxic payload [70].

#### 7.3.1. YL201

This ADC combines a B7H3 antibody with a topoisomerase I inhibitor. In trial NCT05434234, YL201 demonstrated a favorable objective response rate (ORR) in NSCLC patients with advanced disease who had previously received anti-PD-L1 and platinum-based chemotherapy [69].

#### 7.3.2. PD-L1V

A vedotin-based ADC, PD-L1V, was evaluated in a phase 1 study (NCT05208762) for relapsed PD-L1-expressing solid tumors, including NSCLC. This agent showed an ORR of 33% and a disease control rate of 66.7% [71].

#### 7.3.3. Mecbotamab Vedotin (BA3011)

This is a conditionally active biologic AXL-targeting ADC, currently under investigation in trial NCT04681131 for metastatic NSCLC patients with AXL expression, either as monotherapy or in combination with nivolumab [72].

### 7.4. Advanced Biomarkers for Response Prediction and Monitoring

The limitations of traditional PD-L1 testing in BMs have spurred the development of more sophisticated biomarker strategies:

#### 7.4.1. Circulating Tumor DNA (ctDNA)

ctDNA is a highly promising, minimally invasive methodology for predicting immunotherapy response and monitoring disease progression in real-time [73].

#### 7.4.2. Genomic Hypomethylation

Loss of genomic methylation in cell-free DNA has been suggested as a potential predictor of response to immunotherapy in primary tumors. Methylation-based assays on tumoral cell-free DNA can avoid contamination from non-tumoral cells. The methylation profile of primary lung tumors can also predict the risk of BM development and can be detected early in plasma ctDNA [74].

#### 7.4.3. Minimal Residual Disease (MRD)

The CR1STAL study (NCT05198154) demonstrated that 92% of patients with disease progression exhibited ctDNA minimal residual disease positivity after one-year PFS post-immunotherapy, and ctDNA-positive patients had significantly shorter PFS [75].

#### 7.4.4. Cerebrospinal Fluid (CSF) Analysis

CSF analysis is gaining traction as a method to assess treatment response, as it can reflect the immune infiltrates within BMs. CSF cytokine profiles (e.g., IL-6, IL-10, TNF-α) have been identified as immune-related biomarkers correlating with intracranial tumor response to ICIs. CSF analysis is particularly relevant for leptomeningeal disease, where single-cell RNA and cell-free DNA profiling could provide valuable insights into disease dynamics and treatment response [76].

#### 7.4.5. PhenoTIL

This novel computational immune biomarker utilizes machine learning to analyze the TME, predicting treatment outcomes in NSCLC patients. It identifies genes and pathways to categorize risk levels based on treatment, distinguishing between low- and high-risk patients, and holds potential for guiding decisions to avoid chemotherapy [13].

The landscape of immunotherapy for NSCLC BMs is rapidly evolving, shifting beyond single-agent immune checkpoint inhibition and basic PD-L1 testing towards more sophisticated, multi-modal therapeutic strategies and advanced biomarker-driven approaches [13]. This significant trend reflects a growing understanding that the complex and often immunosuppressive brain TME necessitates diverse therapeutic mechanisms. The development of bispecific antibodies, which simultaneously target multiple pathways like PD-1 and VEGF, alongside antibody-drug conjugates that deliver targeted cytotoxic payloads, and novel immune checkpoint modulators (such as LAG-3 and B7-H3 inhibitors), exemplifies this move towards a more comprehensive attack on the tumor [13,65,66,67,68,69,70,71,72,73,74,75,76]. Concurrently, the increasing emphasis on highly sensitive and non-invasive liquid biopsy techniques, including circulating tumor DNA and CSF cytokine profiles, in conjunction with advanced immune profiling of tumor-infiltrating lymphocytes and computational biomarkers like PhenoTIL, indicates a critical shift towards real-time monitoring and highly personalized treatment stratification (Table 5). This progression implies a future where therapeutic decisions for NSCLC BMs will be guided not just by the primary tumor’s characteristics, but by a comprehensive molecular and immunological blueprint derived from the brain lesions themselves or readily accessible biofluids. This individualized approach is expected to allow for more adaptive strategies to overcome resistance mechanisms and optimize patient outcomes in a highly precise manner [72,73,74,75,76].

## 8. Discussion

Immunotherapy has undeniably ushered in a transformative era in the treatment of NSCLC, particularly for patients afflicted with BMs [1,2,3,4]. This marks a profound departure from historical practices where these individuals were frequently excluded from clinical trials due to prevailing assumptions about the CNS’s immune privilege and the challenges of drug delivery across the BBB [6,7,8,9,10].

Current evidence, notably from meta-analyses andsystematic reviews, robustly demonstrates that ICIs, especially agents like pembrolizumab and combination regimens such as nivolumab with ipilimumab, offer significant improvements in OS and achieve meaningful intracranial response rates [8,9,10,11,12]. While the literature coverage of this narrative review was intentionally broad, the inclusion of multiple meta-analyses may have led to partial study overlap. Their findings were interpreted descriptively rather than cumulatively, with attention to consistent trends and methodological differences to reduce overrepresentation of shared datasets. These were retained for their unique methodological and population insights, but future work should compare methodological differences and assess potential bias to better contextualize the evidence. An additional limitation of this narrative review is the heterogeneity in intracranial response assessment across studies, including differences in radiologic response criteria, protocol-specific definitions of iORR/iDCR, and brain imaging follow-up intervals; accordingly, intracranial outcomes were synthesized descriptively and should be interpreted with caution across studies.

This progress underscores a growing confidence in the intracranial activity of ICIs and represents a critical step towards more effective management of this challenging disease manifestation. Ultimately, achieving optimal outcomes for NSCLC BM patients necessitates a highly personalized treatment approach rooted in a comprehensive understanding of each individual’s disease [4,5,6,7,8]. This involves integrating detailed molecular profiling, thorough characterization of the immune landscape within the brain microenvironment, and a careful consideration of clinical factors [35,36].

As immunotherapy continues to redefine the management of NSCLC BMs, several challenges limit the success of ICIs in NSCLC BMs. Response rates to the therapy remain variable; patients on steroid treatment and with active brain metastases are frequently excluded from the studies, which limits the generalizability of existing data. Moreover, additional barriers include biological heterogeneity between primary NSCLC and BMs, variability in PD-L1 expression, which limits the assessment of treatment response. To address these challenges, future research must refine trial design to address current gaps in patient inclusion and response evaluation. Future studies should aim to expand the inclusion of patients with active and steroid-dependent disease and address the unique challenges associated with ICI therapy in this subgroup. Future trials should integrate advanced translational CNS biomarkers and liquid biopsy to better evaluate and predict responses to ICIs. Moreover, there should be an emphasis on optimization of combination strategies by integrating ICIs with radiotherapy or targeted therapies to enhance treatment efficacy.

The complexity of managing brain metastases underscores the indispensable role of a multidisciplinary team dedicated to achieving an optimal balance between efficacy, safety, and quality of life. Collaborative efforts involving neuro-oncologists, radiation oncologists, medical oncologists, and neurosurgeons are paramount to ensure integrated, sequential, and adaptive care that maximizes therapeutic benefit while minimizing toxicity. The future of NSCLC BMs management lies in this synergistic integration of cutting-edge science and collaborative clinical practice.

## 9. Conclusions

Immunotherapy has become an integral part of the treatment landscape for NSCLC and is increasingly being applied to patients with brain metastases. However, most of the available evidence is derived from carefully selected populations, predominantly asymptomatic patients not requiring corticosteroids, as many pivotal ICI trials excluded individuals on >10 mg prednisone-equivalent/day or with uncontrolled intracranial disease. Within these constraints, PD-1/PD-L1 inhibitors—either alone or in combination regimens—have demonstrated clinically meaningful improvements in OS and intracranial responses. Evidence for immune checkpoint inhibitors in NSCLC brain metastases is strongest in selected, asymptomatic patients with minimal or no corticosteroid use, while efficacy in symptomatic or steroid-dependent patients remains uncertain and represents a key evidence gap.

## Figures and Tables

**Table 1:** Summary of Key Meta-Analyses and Systematic Reviews on Immunotherapy for NSCLC BM.

Study (Year)	Design	Number of Studies/Participants	Key Efficacy Outcomes	Key Safety Outcomes	Immunotherapy Agents Evaluated	Reference
Comparative Efficacy and Safety of Immunotherapy (2023)	Systematic Review & NMA	11 studies/1437 participants	OS HR: Pembrolizumab: HR ~0.67; Nivolumab + Ipilimumab: HR ~0.72 iORR effect: Improved ORR reported for embrolizumab (estimate from reported response rates)	No significant AE difference vs. chemotherapy	Pembrolizumab, Nivolumab + Ipilimumab	[10]
Immunotherapy as Single Treatment (META-L-BRAIN Study, 2021)	Systematic Review & Meta-Analysis	12 studies/566 participants	iORR: 16.4% (95% CI 9.8–24) iDCR: 45% (95% CI 33.4–56.9) No benefit difference with vs. without prior RT	Grades 3/4 toxicity rate evaluated	Anti-PD-1 therapy	[12]
BMs Status and Immunotherapy Efficacy in Advanced Lung Cancer (2021)	Systematic Review of RCTs	14 RCTs/9089 participants	OS HR: 0.72 (95% CI 0.58–0.90) PFS HR: 0.68 (95% CI 0.52–0.87) Benefit similar in BM vs. non-BM (OS HR 0.96)	NR, but overall safety benefit implied	Anti-PD-1/PD-L1s	[13]
Goldberg et al., Lancet Oncol. 2016	Prospective	18 participants	iORR: 30–33% iDCR: 56%	NR, but overall safety benefit implied	Pembrolizumab	[14]
Hendriks et al., J Thorac Oncol 2019	Retrospective	271 participants	iORR: 24%	NR, but overall safety benefit implied	Various ICIs	[15]
KEYNOTE-001 (subset), Rizvi et al.	Phase 1	~30 participants	iORR: ~30%	NR, but overall safety benefit implied	Pembrolizumab	[16]
OAK (subset), Peters et al.	Phase 3	65 participants	NR	NR, but overall safety benefit implied	Atezolizumab	[17]
Verzoni et al., 2019	Retrospective	73 participants	iORR: 24% iDCR: 57%	NR, but overall safety benefit implied	Nivolumab	[18]

Abbreviations: OS = Overall Survival; PFS = Progression-Free Survival; HR = Hazard Ratio; ORR = Objective Response Rate; iORR = Intracranial Objective Response Rate; iDCR = Intracranial Disease Control Rate; NR = Not Reported; CI = Confidence Interval; NMA = Network Meta-Analysis; RCT = Randomized Controlled Trial; RT = Radiotherapy; AE = Adverse Event; BM = Brain Metastases; ICI = Immune Checkpoint Inhibitor; PD-1 = Programmed Cell Death Protein 1; PD-L1 = Programmed Death-Ligand 1.

**Table 2:** Comparative Efficacy and Safety Outcomes of Key Immunotherapy Regimens in NSCLC BMs.

Immunotherapy Agent/Regimen	Patient Population (Treated/Untreated BM)	OS (HR)	PFS (HR)	Intracranial ORR	Common Grade 3/4 AEs	Key Findings/Notes	Reference
Pembrolizumab (Monotherapy)	Untreated, asymptomatic BM (Phase 2 trial)	Not reached (2-year OS 34%)	CNS PFS: 2.3 months (95% CI, 1.9-NR)	29.7% (95% CI, 15.9–47.0)	14% treatment-related serious AEs, comparable to other ICI trials; No treatment-related neurological AEs	Most promising ICI for NSCLC BM; intracranial activity in selected untreated patients	[28,29,30,31]
Nivolumab (Monotherapy)	Pretreated, asymptomatic, stable BM (Pooled analyses)	Promising efficacy compared to docetaxel	NR	NR	Acceptable safety	Acceptable safety and promising efficacy in pretreated stable BM.	[32,33]
Nivolumab plus Ipilimumab	Mixed BM population (NMA)	NR	NR	NR	Significant difference in AEs vs. chemotherapy, with more grade 3 and 4 for the immunotherapy cohort	Alternative to improve OS	[20]
Pembrolizumab + Chemotherapy	Mixed BM population (Pooled analyses)	Improved survival irrespective of BM presence	Improved survival irrespective of BM presence	NR	NR	Improved survival regardless of baseline BM	[21]
Anti-PD-1 Therapy (General)	Untreated BM (Systematic Review/Meta-Analysis)	NR	NR	16.4% (95% CI: 9.8–24%)	Grades 3/4 toxicity rate evaluated (specific data not in snippet)	Active as a single treatment for active BM in selected patients	[12]
Immunotherapy (General)	BM patients (Systematic Review of RCTs)	HR: 0.72 (95% CI, 0.58–0.90)	HR: 0.68 (95% CI, 0.52–0.87)	NR	NR	Comparable benefits for BM and non-BM patients.	[13]

Abbreviations: AEs = Adverse Events; BM = Brain Metastases; CI = Confidence Interval; CNS = Central Nervous System; ICI = Immune Checkpoint Inhibitor; NMA = Network Meta-Analysis; NR = Not Reported; NSCLC = Non-Small Cell Lung Cancer; OS = Overall Survival; PD-1 = Programmed Cell Death Protein 1; PFS = Progression-Free Survival.

**Table 3:** PD-L1 Expression and Its Correlation with Immunotherapy Outcomes in NSCLC BMs.

Study/Source	PD-L1 Cutoff/Assessment Method	Correlation with OS/PFS/ORR	Concordance Data (Primary vs. BM)	Challenges Noted	Reference
Tonse et al. (2020). “Systematic review and meta-analysis of PD-L1 expression discordance between primary tumor and lung cancer BMs“ for the 19% discordance rate	PD-L1 expression, PD-L1 levels, TPS	Higher PD-L1 scores associated with better OS	19% disagreement between primary lung tumors and BM (BM generally lower)	Discordance between primary and BM; Not a definitive biomarker	[51]
Camy et al. (2020). “BM PD-L1 and CD8 expression is dependent on primary tumor type and its PD-L1 and CD8 status,” which highlights the complex and sometimes contradictory nature of PD-L1 expression in BMs	High (≥50%) vs. Low (≤1%) PD-L1 expression	No statistically significant association between high PD-L1 and lower progression/development of BM	NR	Contradictory findings; Small sample size for high PD-L1 group	[52]
Davis et al. (2017). “PD-L1 as a biomarker in NSCLC: challenges and future directions,” a review article that details the issues with different assays and scoring methods	PD-L1 positivity, influenced by assay, algorithm, cutoff, and metastatic site	PD-L1 expression, used to predict responses, but not a definitive biomarker	NR	PD-L1 positivity influenced by assay/site; Need for novel predictive biomarkers beyond PD-L1	[53]
Regulatory guidelines and clinical trial protocols (e.g., FDA-approved materials for KEYTRUDA) that define TPS and CPS for patient selection	TPS (percentage of viable tumor cells with membrane staining); CPS (number of PD-L1-staining cells relative to all viable tumor cells). Min 100 viable tumor cells	General guidelines for KEYTRUDA indications (not specific to BM)	NR	Methodological variations in assessment	[46,47]
Studies such as Batur et al. (2020) and Camy et al. (2020) that show a range of concordance rates and discuss the unique TME of the brain	<1%, 1–49%, ≥50% (Immunohistochemistry (IHC) staining)	Low PD-L1 expression associated with better PFS and OS (contradictory to the general trend)	62.5% to 81% concordance reported; Brain metastatic tumors often have less PD-L1 expression	Discordance/heterogeneity; Brain BM lesions exhibit more immunosuppressive TME	[42,52]
Batur et al. (2020). “Concordance of PD-L1 expression and CD8+ TIL intensity between NSCLC and synchronous BMs,” which provides these specific concordance percentages	<1%, 1–49%, ≥50% (IHC on tumor cells)	NR	High concordance of PD-L1 expression in neoplastic cells between primary NSCLC and synchronous BMs	PD-L1 expression on TILs not strongly correlated; CD8+ TIL intensity only 54.16% concordant	[42]
Clinical reviews and commentaries on the challenges of treating BMs and the need for new biomarkers. This is a common theme in oncology literature	PD-L1 TPS	Unclear if extracranial PD-L1 predicts BM benefit	Low level of concordance between primary and metastatic sites suggested	Difficulty in obtaining BM tissues for testing; Need for further studies on BM-specific PD-L1	[54,55]

Abbreviations: ADC = Antibody-Drug conjugate; AEs = Adverse Events; BBB = Blood–Brain Barrier; BM/BMs = Brain Metastases; CI = Confidence Interval; CNS = Central Nervous System; CPS = Combined Positive Score; CTLA-4 = Cytotoxic T-Lymphocyte-Associated Protein 4; HR = Hazard Ratio; ICIs = Immune Checkpoint Inhibitors; iDCR = Intracranial Disease Control Rate; IHC = Immunohistochemistry; iORR = Intracranial Objective Response Rate; N = Number of Patients; NMA = Network Meta-Analysis; NR = Not Reported; NSCLC = Non-Small Cell Lung Cancer; ORR = Objective/Overall Response Rate; OS = Overall Survival; PD-1 = Programmed Cell Death Protein 1; PD-L1 = Programmed Death-Ligand 1; PFS = Progression-Free Survival; RCT = Randomized Controlled Trial; RT = Radiotherapy; TIL = Tumor Infiltrating Lymphocyte; TME = Tumor Microenvironment; TPS = Tumor Proportion Score.

**Table 4:** Overview of Patient Inclusion/Exclusion Criteria for BMs in Key Immunotherapy Trials.

Trial Name	Immunotherapy Type	CNS Disease Criteria (Inclusion/Exclusion)	Steroid Policy	Key Patient Population Characteristics	Reference
Goldberg et al.	Pembrolizumab monotherapy	Inclusion: At least one asymptomatic BM 5–20 mm, not previously treated or progressing after radiotherapy	Not requiring steroids	Stage IV non-oncogene driven, ICI-naive NSCLC; PD-L1 TPS ≥ 1% (*n* = 37) or 0% (*n* = 5)	[54]
Hellman et al., 2017	Nivolumab monotherapy	Inclusion: At least one asymptomatic and untreated BM up to 30 mm, up to four BMs	Patients were excluded if they had active cerebral edema requiring corticosteroid therapy.	NSCLC, with at least one prior systemic therapy	[27]
PERLA et al.	Dostarlimab + Chemo vs. Pembrolizumab + Chemotherapy	Exclusion: Known active BM and/or leptomeningeal metastases. Inclusion (conditional): Treated and stable BM (neurologically stable ≥ 2 weeks, off corticosteroids ≥ 3 days); Untreated, asymptomatic BM (no neurological symptoms, no corticosteroids, no/minimal edema, no lesions > 1.5 cm)	Patients were excluded if they were receiving systemic corticosteroids at immunosuppressive doses, defined as >10 mg/day prednisone-equivalent (or >2 mg/day dexamethasone-equivalent) within 7–14 days prior to treatment initiation, or if they required ongoing systemic immunosuppression for any reason	Metastatic non-squamous NSCLC, no EGFR/ALK/ROS-1/BRAF V600E mutations; Documented PD-L1 status	[63]
KEYNOTE-189	Pembrolizumab + Chemotherapy	Exclusion: Known active CNS metastases and/or carcinomatous meningitis	Patients were excluded if they were receiving systemic corticosteroids at immunosuppressive doses, defined as >10 mg/day prednisone-equivalent (or >2 mg/day dexamethasone-equivalent) within 7–14 days prior to treatment initiation, or if they required ongoing systemic immunosuppression for any reason	Stage IV non-squamous NSCLC; No prior systemic treatment for advanced NSCLC; No EGFR/ALK aberrations	[59]
CheckMate 227	Nivolumab, Nivolumab + Ipilimumab, or Nivolumab + Platinum Chemotherapy	Exclusion: Untreated CNS metastases	Patients were excluded if they were receiving systemic corticosteroids at immunosuppressive doses, defined as >10 mg/day prednisone-equivalent (or >2 mg/day dexamethasone-equivalent) within 7–14 days prior to treatment initiation, or if they required ongoing systemic immunosuppression for any reason.	Stage IV or recurrent NSCLC; Chemotherapy-naive; PD-L1 IHC testing required	[20]
NCT02886585 (Phase II)	Pembrolizumab monotherapy	Inclusion: Untreated asymptomatic BM or progressive asymptomatic BM ≥10 mm or cytology-positive neoplastic meningitis	Patients were excluded if they were receiving systemic corticosteroids at immunosuppressive doses, defined as >10 mg/day prednisone-equivalent (or >2 mg/day dexamethasone-equivalent) within 7–14 days prior to treatment initiation, or if they required ongoing systemic immunosuppression for any reason	Solid tumors (NSCLC, melanoma, etc.); Measurable CNS disease (≥5 mm)	[64]
SUNRAY-01	Olomorasib + Pembrolizumab +/− Chemotherapy	Exclusion: Known active CNS metastases and/or carcinomatous meningitis.	NR	KRAS G12C-Mutant NSCLC; Stage IIIB-IV; Known PD-L1 expression	[62]
CodeBreaK 202	Sotorasib + Chemo vs. Pembrolizumab + Chemotherapy	Exclusion: Symptomatic (treated or untreated) BMs.	NR	Non squamous Stage IV/advanced IIIB/IIIC NSCLC; KRAS p.G12C mutation; PD-L1 negative	[61]
IMpower150	Atezolizumab + Bevacizumab + Chemotherapy	Exclusion: Active or untreated CNS metastases	NR	Stage IV non-squamous NSCLC; No prior treatment for Stage IV NSCLC; Known PD-L1 status	[60]

Abbreviations: AE = Adverse Event; ALK = Anaplastic Lymphoma Kinase; BM = Brain Metastasis; BRAF = B-Raf Proto-Oncogene; BRAF V600E = BRAF Valine-to-Glutamic Acid Substitution at Codon 600; CNS = Central Nervous System; CSF = Cerebrospinal Fluid; EGFR = Epidermal Growth Factor Receptor; ICI = Immune Checkpoint Inhibitor; IHC = Immunohistochemistry; KRAS = Kirsten Rat Sarcoma Viral Oncogene Homolog; KRAS G12C = KRAS Glycine-to-Cysteine Substitution at Codon 12; NR = Not Reported; NSCLC = Non-Small Cell Lung Cancer; PD-1 = Programmed Cell Death Protein 1; PD-L1 = Programmed Death-Ligand 1; ROS1 = ROS Proto-Oncogene 1, Receptor Tyrosine Kinase; TPS = Tumor Proportion Score.

**Table 5:** Emerging Biomarkers and Novel Therapeutic Strategies in NSCLC BMs Immunotherapy.

Biomarker/Strategy	Mechanism/Target	Potential Application	Current Status/Key Findings	Reference
***Emerging Biomarkers***				
PhenoTIL	Computational immune biomarker analysis of TME via machine learning.	Predicts treatment outcomes, categorizes risk, and potentially guides chemotherapy avoidance	Predicts outcomes in NSCLC, distinguishes risk levels	[13]
CtDNA (Genomic Hypomethylation)	Loss of genomic methylation in cell-free DNA.	Predicts ICI response; Predicts BM risk; Real-time monitoring	Potential predictor of ICI response; Methylation profiles predict BM risk in plasma ctDNA	[73]
CtDNA (MRD)	ctDNA positivity after treatment.	Predicts shorter PFS post-immunotherapy	CR1STAL study (NCT05198154) showed that ctDNA MRD positivity correlated with shorter PFS	[75]
CSF Analysis	CSF cytokine profiles (IL-6, IL-10, TNF-α), single-cell RNA, and cell-free DNA profiling.	Assesses treatment response; Recapitulates BM immune infiltrates; Relevant for leptomeningeal disease	CSF cytokine profiles correlate with intracranial tumor response; the initial inflammatory response (IFN-γ) suggested prognostic value	[76]
Tumor-Reactive CD8+ TILs	Expression of specific markers (CD39, CD103, CXCL13, PD-1, TIM-3).	Potential biomarkers for ICI response; Guides immunotherapy via transcriptomic profiling	Identified via single-cell RNA sequencing as potential biomarkers	[42]
***Novel Therapeutic Strategies***				
LAG-3 Inhibitors (e.g., Relatlimab)	Targets LAG-3 immune checkpoint	Enhances anti-tumor immunity in combination	RELATIVITY-104 (NCT04623775) showed benefit with nivolumab + relatlimab + chemotherapy	[65]
B7-H3 Targeting	Targets B7-H3, which promotes immune suppression and remodels vasculature	Reverts vasculature leakiness, enhances CD8+ T-cell infiltration	Preclinical promise in mouse models	[66]
Bispecific Antibodies (e.g., Cadonilimab)	Targets two distinct antigens (e.g., PD-1 and VEGF)	Enhanced efficacy, broader anti-tumor activity	Cadonilimab (AK104) in COMPASSION-01 trial (NCT03261011) for metastatic NSCLC	[68]
Bispecific Antibodies (e.g., Ivonescimab)	Targets PD-1 and VEGF simultaneously	PFS benefit in BM patients	HARMONi-2 trial (NCT05499390) suggests PFS benefit vs. pembrolizumab in NSCLC BM	[69]
ADCs (e.g., YL201)	B7H3 antibody + topoisomerase I inhibitor	Targeted cytotoxic payload delivery	Trial NCT05434234 showed good ORR in previously treated NSCLC	[70]
ADCs (e.g., PD-L1V)	Vedotin-based ADC targeting PD-L1	Targeted cytotoxic payload delivery to PD-L1 expressing cells	Phase 1 study (NCT05208762) showed 33% ORR, 66.7% DCR in relapsed PD-L1-expressing solid tumors	[71]
ADCs (e.g., Mecbotamab vedotin)	AXL-targeting ADC	Targeted therapy for AXL-expressing metastatic NSCLC	Investigated in trial NCT04681131 alone or with nivolumab	[72]

Abbreviations: ADC= Antibody–Drug Conjugate); AXL = AXL Receptor Tyrosine Kinase; B7H3 = B7 Homolog 3 Immune Checkpoint Molecule; BM = Brain Metastasis; CSF = Cerebrospinal Fluid; ctDNA = Circulating Tumor DNA; CXCL13 = C-X-C Motif Chemokine Ligand 13; DCR = Disease Control Rate; IFN-γ = Interferon Gamma; ICI = Immune Checkpoint Inhibitor; IL-6 = Interleukin 6; IL-10 = Interleukin 10; LAG-3 = Lymphocyte Activation Gene 3; MRD = Minimal Residual Disease; NSCLC = Non–Small Cell Lung Cancer; ORR = Objective Response Rate; PD-1 = Programmed Cell Death Protein 1; PD-L1 = Programmed Death-Ligand 1; PFS = Progression-Free Survival; TIL/TILs = Tumor-Infiltrating Lymphocyte(s); TIM-3 = T-Cell Immunoglobulin and Mucin-Domain Containing-3; TNF-α = Tumor Necrosis Factor Alpha; TME = Tumor Microenvironment; VEGF = Vascular Endothelial Growth Factor.

## Data Availability

The original contributions presented in this study are included in the article. Further inquiries can be directed to the corresponding author.

## Abbreviations

The following abbreviations are used in this manuscript:

ADC	Antibody-Drug conjugate
AEs	Adverse Events
ALK	Anaplastic Lymphoma Kinase
AXL	AXL Receptor Tyrosine Kinase
B7H3	B7 Homolog 3 Immune Checkpoint Molecule
BBB	Blood–Brain Barrier
BMs	Brain Metastases
BRAF	B-Raf Proto-Oncogene
BRAF V600E	BRAF Valine-to-Glutamic Acid Substitution at Codon 600
CI	Confidence Interval
CNS	Central Nervous System
CPS	Combined Positive Score
ctDNA	Circulating Tumor DNA
CTLA-4	Cytotoxic T-Lymphocyte-Associated Protein 4
CXCL13	C-X-C Motif Chemokine Ligand 13
EGFR	Epidermal Growth Factor Receptor
HR	Hazard Ratio
ICIs	Immune Checkpoint Inhibitors
iDCR	Intracranial Disease Control Rate
IFN-γ	Interferon Gamma
IHC	Immunohistochemistry
iORR	Intracranial Objective Response Rate
KRAS	Kirsten Rat Sarcoma Viral Oncogene Homolog
KRAS G12C	KRAS Glycine-to-Cysteine Substitution at Codon 12
LAG-3	Lymphocyte Activation Gene 3
MRD	Minimal Residual Disease Rate
N	Number of Patients
NMA	Network Meta-Analysis
NR	Not Reported
NSCLC	Non-Small Cell Lung Cancer
ORR	Overall Response Rate
OS	Overall Survival
PD-1	Programmed Deathcell death protein 1
PD-L1	Programmed Death-Ligand 1
PFS	Progression-Free Survival
RCT	Randomized Controlled Trial
ROS1	ROS Proto-Oncogene 1, Receptor Tyrosine Kinase
RT	Radiotherapy
TIL	Tumor Infiltrating Lymphocyte
TIM-3	T-Cell Immunoglobulin and Mucin-Domain Containing-3
TME	Tumor Microenvironment
TPS	Tumor Proportion Score
VEGF	Vascular Endothelial Growth Factor
